# Assessing Changes in Symptoms of Depression and Anxiety During Four Weeks of Cannabis Abstinence Among Adolescents

**DOI:** 10.3389/fpsyt.2021.689957

**Published:** 2021-07-01

**Authors:** Megan E. Cooke, Jodi M. Gilman, Erin Lamberth, Natali Rychik, Brenden Tervo-Clemmens, A. Eden Evins, Randi M. Schuster

**Affiliations:** ^1^Center for Addiction Medicine, Department of Psychiatry, Massachusetts General Hospital, Boston, MA, United States; ^2^Harvard Medical School, Boston, MA, United States

**Keywords:** cannabis, cannabis abstinence, depression, anxiety, contingency management, youth, adolescents

## Abstract

**Background:** Cannabis use is prevalent among adolescents, and many report using in attempts to alleviate negative mood and anxiety. Abstinence from substances such as alcohol and tobacco has been reported to improve symptoms of anxiety and depression. Few studies have examined the effect of cannabis abstinence on symptoms of anxiety and depression.

**Objective:** To test the effect of 4 weeks of continuous cannabis abstinence on depressive and anxious symptoms.

**Methods:** Healthy, non-treatment seeking adolescents who used cannabis at least weekly (*n* = 179) were randomized to either 4 weeks of cannabis abstinence achieved through a contingency management paradigm (CB-Abst) or cannabis use monitoring without an abstinence requirement (CB-Mon). Abstinence was assessed by self-report verified with quantitative assay of urine for cannabinoids. Anxiety and depressive symptoms were assessed weekly with the Mood and Anxiety Symptom Questionnaire (MASQ).

**Results:** Symptoms of depression and anxiety decreased throughout the study for all participants (MASQ-AA: stnd beta = −0.08, *p* = 0.01, MASQ-GDA: stnd beta = −0.11, *p* = 0.003, MASQ-GDD: stnd beta = −0.08, *p* = 0.02) and did not differ significantly between randomization groups (*p*'s > 0.46). Exploratory analyses revealed a trend that abstinence may be associated with greater improvement in symptoms of anxiety and depression among those using cannabis to cope with negative affect and those with potentially hazardous levels of cannabis use.

**Conclusions:** Among adolescents who use cannabis at least weekly, 4 weeks of cannabis abstinence was not associated with a significant change in anxiety or depressive symptoms compared to continued use. For recreational cannabis users who may be concerned about reducing their use for fear of increased symptoms of anxiety and depression, findings suggest that significant symptom worsening may not occur within the first 4 weeks of abstinence. Further studies are needed in clinical populations where anxiety and depression symptoms are measured more frequently and for a longer period of abstinence. Future studies are also needed to determine whether there are subgroups of adolescents who are uniquely impacted by sustained cannabis abstinence.

## Introduction

More high school students use cannabis daily than any other substance (1) and perception of cannabis-related harm among adolescents, a key indicator of uptake of use, is at its lowest level in nearly four decades (1, 2) while cannabis potency has significantly increased (3). Youth cannabis exposure is growing with expanding commercial recreational cannabis markets across the United States, that impose few potency limits and derive the majority of profit from products such as candies that appeal to youth (4, 5).

Many people who use cannabis endorse using cannabis in an attempt to cope with stress, anxiety, and depression (6–8), and this is true for youth who are recent or frequent users (7, 9). Using cannabis to cope with negative emotions, however, has been associated with more persistent use, cannabis-related problems, cannabis dependence, and psychiatric dysfunction (9–12). Thus, though many cite alleviation of mood symptoms as a primary motive for cannabis use, there is reason to believe cannabis use may *in fact* exacerbate these symptoms. Cross-sectional studies report associations between cannabis use and higher odds of depression (ORs: 1.2–1.7) (13–16), and longitudinal studies show elevated rates of subsequent depression and anxiety in young cannabis users, even after adjustment for baseline covariates. There is an urgent need to understand the effect of cannabis use and its discontinuation on symptoms of depression and anxiety, particularly in adolescents.

Randomized controlled trials of cannabis abstinence can help clarify the effect of cannabis on depression and anxiety symptoms. By randomizing cannabis users to a period of abstinence, we can examine the potential unique effect of cannabis abstinence on depression and anxiety symptoms regardless of an individual's baseline symptoms or motivations for substance use. Abstaining from other recreational drugs (alcohol, tobacco) is associated with clinically significant improvement in depression, anxiety, and perceived stress (17, 18). It is important to understand the impact of cannabis abstinence on these symptoms. Due to the similar symptomatology (e.g., amotivation, anhedonia) (19, 20), mechanisms (e.g., dysregulation of CB1 receptors) (21), and neurocircuitry (e.g., abnormalities in the reward structures and limbic system) (22–27) shared by cannabis use and mood disorders, it is anticipated that symptoms of anxiety and depression would fluctuate during cannabis abstinence as seen with other substances. The magnitude, direction, and duration of psychiatric symptom fluctuation is essential information for clinicians to inform the extent to which they should monitor depression and anxiety during an abstinence attempt or advise on mood and/or anxiety benefits associated with abstinence.

In this study, we randomized adolescent cannabis users to 4 weeks of either frequent monitoring with incentives provided for completion of assessments without requirement for abstinence (monitoring) or monetary incentives contingent upon continuous, biochemically verified cannabis abstinence (contingency management). Contingency management (CM) using financial incentives has been shown to reliably induce verified abstinence from many types of drugs, including cannabis (28–39). The goal of this study was to understand the effect of cannabis abstinence on depression and anxiety symptoms in youth who use cannabis at least weekly. Based on previous literature, we hypothesized that youth who discontinued frequent cannabis use would have a greater reduction in symptoms of depression and anxiety over 4 weeks of abstinence compared to youth who continued frequent cannabis use.

## Methods

### Participants

Participants for the present study are part of an ongoing clinical trial examining the effects of cannabis abstinence on cognition (NCT03276221). Participants were recruited from the community as well as middle and high schools in the greater Boston area. Participants were non-treatment seeking, medically healthy, at least weekly cannabis users who were willing to abstain from cannabis use for 4 weeks. Additional eligibility criteria included English fluency and no history of severe developmental delays.

### Procedures

Prior to beginning study procedures, written informed consent was obtained for all participants ages 18 years and older, and written parental consent and participant assent were obtained for participants under the age of 18 years. All study procedures were approved by the Partners Healthcare Human Subjects Committee. A detailed description of study procedures has been documented elsewhere (40–43). Briefly, at the baseline visit participants were randomized to 4 weeks of cannabis abstinence using an escalating financial incentive structure (contingency management; CB-Abst) or 4 weeks of monitoring with no abstinence requirement (CB-Mon). Randomization was stratified by sex (male or female), age (13–16 or 17 and older), and frequency of cannabis use (1 day per week or >1 day per week). CB-Abst and CB-Mon completed in person visits to verify abstinence at baseline and at an average of 2 days (visit 2), 3 days (visit 3), 1 week (visit 4), 2 weeks (visit 5), 3 weeks (visit 6) and 4 weeks (visit 7) after baseline. For these analyses, we evaluated data collected at baseline, and weeks one through four (visit 1 and 4–7).

### Assessments

Anxiety and depression symptoms were assessed weekly using the Mood and Anxiety Symptom Questionnaire (MASQ) Short Form (44, 45) which has four subscales; general distress anxious symptoms (GDA), anxious arousal (AA), general distress depressive symptoms (GDD), and anhedonic depression (AD). Higher scores indicate greater severity of symptoms. Motives for cannabis use were assessed at baseline using the Marijuana Motives Measure (MMM) (46). Of the four subscales of the MMM, we focused on the coping motives subscale for the current study, which consists of five questions assessing the extent to which participants used cannabis to cope with negative emotions and experiences (e.g., “To forget my worries,” “Because it helps me when I feel depressed or nervous”). Frequency of cannabis use over the past 90 days was assessed at baseline using a modified Timeline Follow-Back interview (47). The Cannabis Use Disorders Identification Test-Revised (CUDIT-R) was used to assess the severity of cannabis use at baseline (48). To examine differences in subjective experiences of withdrawal between groups, cannabis withdrawal symptoms were assessed at every visit in both the CB-Abst and CB-Mon groups using the intensity subscale of the Cannabis Withdrawal Scale [CWS-I; (49)].

Urine samples were collected at every visit from all participants and cannabis was assessed qualitatively, via immunoassay rapid dip drug test (RDDT; Medimpex United Inc.), and quantitatively, via liquid chromatography-tandem mass spectrometry (Dominion Diagnostics, North Kingstown, Rhode Island, USA). Self-reported cannabis abstinence was biochemically verified in the CB-Abst group by progressively decreasing concentrations of creatinine adjusted 11-nor-9-carboxy-Δ9-tetrahydrocannabinol [CN-THCCOOH, (50)).

### Analytic Approach

Participants randomized to CB-Abst who did not meet abstinence criteria during the first week of the 4-week abstinence period were given the opportunity to recommit to abstinence and if subsequently successful were included in this study. Those that recommitted to abstinence but did not meet abstinence criteria after the first week of the 4-week abstinence period were excluded from the present study. Participants in the CB-Abst group who met abstinence criteria during the first week but not for the entire 4-week period were censored at the point of resumption of use. We compared the CB-Abst and CB-Mon groups on baseline characteristics using *t*-tests and chi-square tests as appropriate. To assess change in withdrawal, we computed change scores in CWS-I from baseline for each weekly time point. We tested the difference in CWS-I between groups using *t*-tests. For the primary analyses, examining change in MASQ scores by group, time was analyzed as a continuous measure representing days from baseline (date of randomization). Linear mixed effects models were used to test the effects of randomization group, time, and their interaction on the MASQ subscales in the full sample. We also explored effects in two subsets: one in those participants who endorsed frequently using cannabis to cope with negative emotions (MMM coping subscale score ≥ 3; *n* = 40) and one in participants with probable cannabis use disorder (CUDIT score ≥ 12; *n* = 116). Age, sex, Hispanic ethnicity, baseline CN-THCCOOH and baseline MASQ score were included as fixed effects covariates. Participant was included as a random effect on both the intercept and the time since baseline slope. All models were estimated with the lme4 package in R (version 4.0.2). Significance values were computed using the lmerTest package (51).

## Results

### Participant Characteristics

See Table 1 for descriptive statistics. There were no differences between the CB-Abst and CB-Mon groups except on number of Hispanic participants, baseline CN-THCCOOH, baseline MASQ-GDA, baseline MASQ-GDD, and baseline MMM-Coping scores. While the sample was ascertained from the community, 64.8% of participants reported CUDIT scores ≥12 at baseline, indicating a potential cannabis use disorder.

**Table 1:** Participant characteristics by CB-Abst and CB-Mon groups.

**Measure**	**CB-Abst**	**CB-Mon**
*N*	101	78
Age	19.7 (2.0)	19.2 (2.3)
Sex - female	45 (44.6%)	35 (44.8%)
Race - nonwhite	33 (32.7%)	37 (47.4%)
Ethnicity – Hispanic^*^	9 (8.9%)	17 (21.8%)
Age of first cannabis use	15.4 (1.9)	15.4 (2.0)
Days per week of cannabis use	4.6 (2.0)	4.5 (2.2)
Baseline CN-THCCOOH^*^	150.2 (187.6)	294.6 (536.5)
Baseline CUDIT-R	14.0 (5.6)	13.6 (5.1)
Baseline CWS - I	33.6 (24.6)	30.3 (17.4)
Baseline MASQ – GDA^*^	19.7 (6.6)	17.4 (6.1)
Baseline MASQ - AA	24.8 (6.7)	24.3 (7.0)
Baseline MASQ – GDD^*^	23.1 (9.2)	19.9 (8.0)
Baseline MASQ - AD	58.7 (13.1)	56.3 (11.4)
Baseline MMM-Coping^*^	2.3 (1.0)	2.05 (0.8)

*For continuous measures numbers represent the mean (SD); for categorical measures numbers represent n (%). CUDIT, Cannabis Use Disorder Identification Test; CWS-I, Cannabis Withdrawal Scale - Intensity subscale; MASQ, Mood and Anxiety Symptoms Questionnaire; GDA, general distress anxious symptoms; AA, anxious arousal; GDD, general distress depression symptoms; AD, anhedonic depression; MMM, Marijuana Motives Measure*.

* *p < 0.05*.

### Abstinence Rates in CB-Abst Group

**Figure 1:**
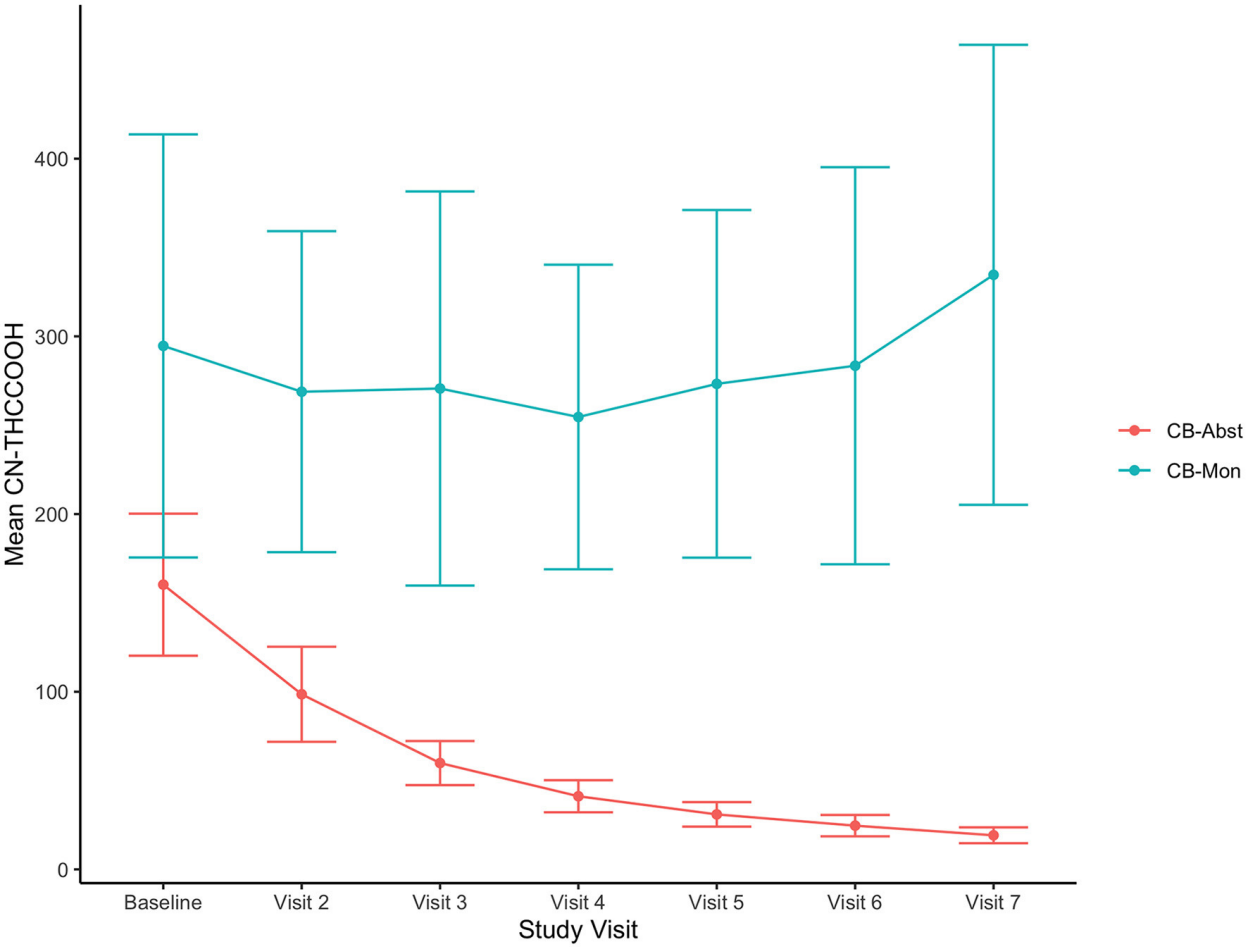
Decreasing CN-THCCOOH concentrations in CB-Abst. Figure shows average urine creatine adjusted 11-nor-9-carboxy-Δ9-tetrahydrocannabinol (CN-THCCOOH) concentration (ng/mL) and confidence intervals at each study visit for CB-Abst and CB-Mon groups.

Of the participants randomized to the CB-Abst group (*n* = 112), 76.8% (*n* = 86) were abstinent for the full 4 weeks. Four participants resumed use within the first week of abstinence but per study protocol were allowed to recommit to abstinence and were successfully abstinent for the remainder of the study, totaling 90 participants with ~4 weeks of abstinence (80%). Data from an additional 11 participants were censored from these analyses due to resumption of cannabis use between weeks one through four; three of whom used between weeks one and two, five of whom used between weeks two and three, and three of whom used between weeks three and four. Participants in the CB-Abst group who resumed cannabis use, withdrew consent or were lost to follow up (*n* = 22) were more frequent cannabis users (5.5 days per week vs. 4.5 days per week, *p* = 0.03) and had significantly higher CUDIT scores (17.2 vs. 13.7, *p* = 0.01) than participants who remained in the study and remained abstinent (*n* = 93). Participants in the CB-Abst group who remained abstinent did not significantly differ on baseline MASQ scores from participants in the CB-Abst group who did not remain abstinent or were lost to follow up (*p*'s > 0.18). None of the CB-Mon participants were voluntarily abstinent for the full 4 weeks. Comparing cannabis use at the baseline visit to the week four visit in the CB-Mon group, we found no significant change in the number of days they used (M = 0.02, sd = 2.3, *p* = 0.95) or the number of grams used per week (M = −0.54, sd = 5.6, *p* = 0.45) but a significant increase in the number of times/sessions per week they used (M = 1.99, sd = 6.6, *p* = 0.02). As demonstrated previously in this sample (42), urine metabolites decreased in the CB-Abst group and did not change in the CB-Mon group (see Figure 1).

### Change in Withdrawal Over Time

CB-Abst had a greater change in CWS-I scores from baseline than CB-Mon 1 week after randomization (diff in means = 5.96, *p* < 0.001). There was no difference between groups in CWS-I change from baseline at 2, 3, and 4 weeks post randomization (*p*'s > 0.09).

### Change in Mood Symptoms During Abstinence

**Figure 2:**
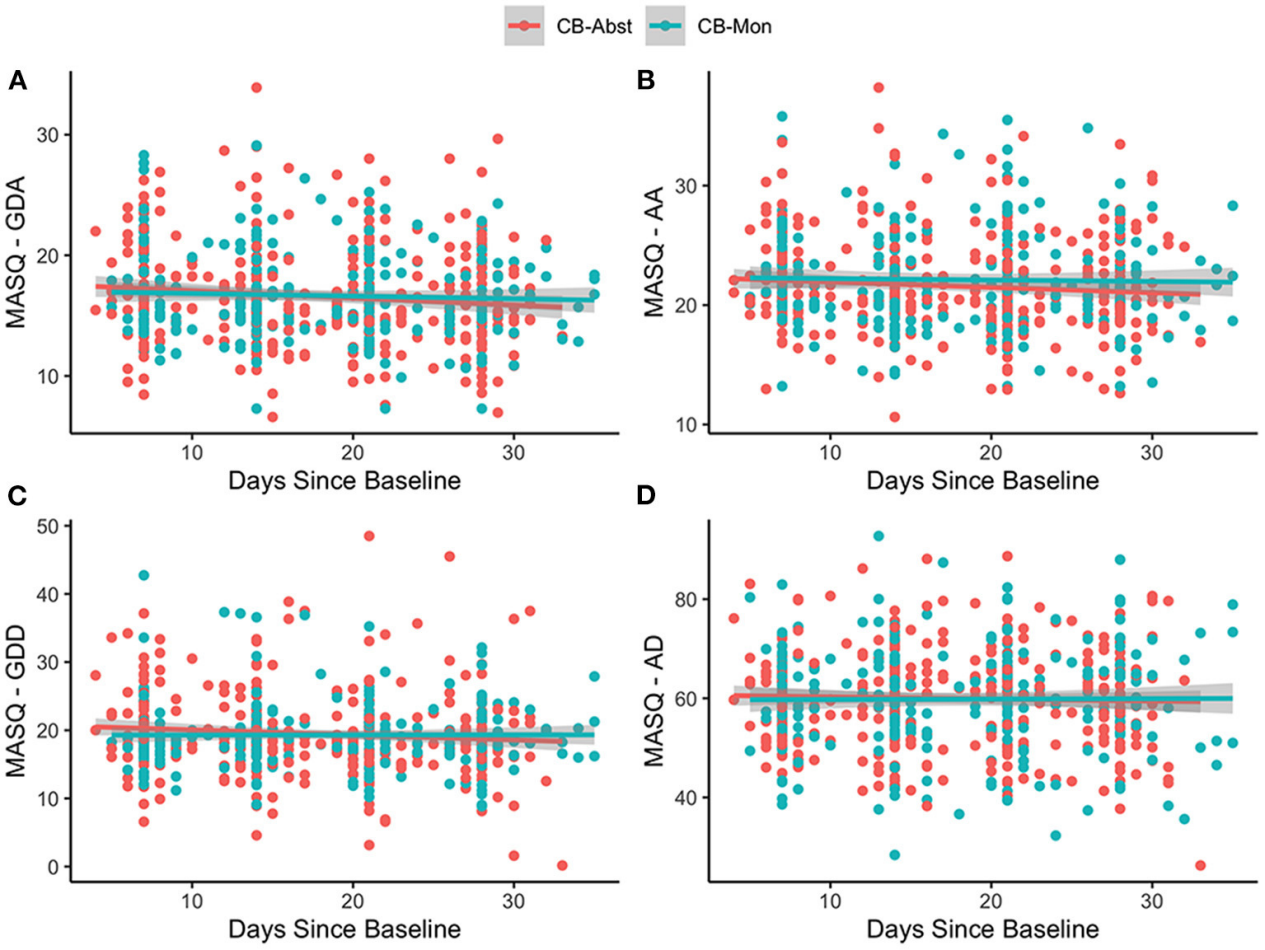
MASQ scores by group over time. Figures show the individual observations of each MASQ subscale which have been adjusted for age, sex and baseline MASQ subscale as well as predictive slopes across time by randomization group; CB-Abst in coral and CB-Mon in teal. Supplementary Figure 1 displays the mean group differences with confidence intervals between CB-Abst and CB-Mon by visit. **(A)** age: stnd beta = −0.03, *p* = 0.58, sex: stnd beta = 0.01, *p* = 0.96, ethnicity: stnd beta = 0.08, *p* = 0.57, baseline CN-THCCOOH: stnd beta = −0.01, *p* = 0.81, baseline MASQ-GDA: stnd beta = 0.65, *p* < 0.001, time: stnd beta = −0.11, *p* = 0.003, group: stnd beta = −0.02, *p* = 0.86, time by group interaction: stnd beta = 0.08, *p* = 0.18. **(B)** age: stnd beta = −0.04, *p* = 0.41, sex: stnd beta = 0.002, *p* = 0.98, ethnicity: stnd beta = 0.07, *p* = 0.64, baseline CN-THCCOOH: stnd beta = −0.006, *p* = 0.91, baseline MASQ-AA: stnd beta = 0.72, *p* < 0.001, time: stnd beta = −0.08, *p* = 0.01, group: stnd beta = 0.07, *p* = 0.51, time by group interaction: stnd beta = 0.07, *p* = 0.20. **(C)** age: stnd beta = −0.01, *p* = 0.84, sex: stnd beta = 0.11, *p* = 0.27, ethnicity: stnd beta = 0.04, *p* = 0.75, baseline CN-THCCOOH: stnd beta = 0.01, *p* = 0.84, baseline MASQ-GDD: stnd beta = 0.66, *p* < 0.001, time: stnd beta = −0.08, *p* = 0.02, group: stnd beta = −0.07, *p* = 0.46, time by group interaction: stnd beta = 0.08, *p* = 0.12. **(D)** age: stnd beta = −0.05, *p* = 0.36, sex: stnd beta = −0.03 *p* = 0.73, ethnicity: stnd beta = 0.13, *p* = 0.32, baseline CN-THCCOOH: stnd beta = 0.01, *p* = 0.84, baseline MASQ-AD: stnd beta = 0.69, *p* < 0.001, time: stnd beta = −0.03, *p* = 0.34, group: stnd beta = −0.04, *p* = 0.72, time by group interaction: stnd beta = 0.03, *p* = 0.53.

There was no significant main effect of age, sex, ethnicity, or baseline CN-THCCOOH levels in any of the models (*p*'s > 0.27). There was a significant main effect of baseline symptoms for each MASQ subscale (GDA, AA, GDD, AD) (stnd beta = 0.65–0.72, all *p*'s < 0.001), suggesting that baseline mood and anxiety symptoms predicted average mood and anxiety symptoms across all study visits. There was no main effect of randomization group on any of the MASQ subscales (*p*'s > 0.46) during the study period, suggesting that overall anxiety and depression symptoms did not differ between CB-Abst and CB-Mon. There was a significant effect of days since baseline on MASQ-GDA (stnd beta = −0.11, *p* = 0.003), MASQ-AA (stnd beta = −0.08, *p* = 0.01), and MASQ-GDD (stnd beta = −0.08, *p* = 0.02), such that symptoms decreased over time on average across randomization groups. There was no interaction between randomization group and days since baseline on any of the MASQ subscales (*p*'s > 0.12), suggesting changes in mood and anxiety symptoms did not significantly differ as a function of cannabis abstinence (see Figure 2).

### Mood Changes Among Those Who Use Cannabis to Cope With Mood

With the exception of baseline symptoms (stnd beta = 0.44–0.79, *p*'s < 0.001), no other covariates or randomization group were associated with MASQ scores in a subgroup of participants who endorsed using cannabis to cope with negative emotions on half or more of the times they used (*n* = 40; *p*'s > 0.15). There was a significant main effect of days since baseline on MASQ-GDA (stnd beta = −0.17, *p* = 0.02), MASQ-GDD (stnd beta = −0.16, *p* = 0.03), and MASQ-AD (stnd beta = −0.24, *p* = 0.006), such that scores on these scales decreased over time on average across randomization groups. Within this subgroup, there was a trend toward an interaction effect of randomization group and days since baseline on MASQ-AD (*p* = 0.056), with greater declines in scores over time in the CB-Abst group compared to the CB-Mon group. There were no significant interaction effects on any of the other MASQ subscales (*p*'s > 0.22).

### Mood Changes Among Those With Problem Cannabis Use

In a subgroup of participants who reported baseline CUDIT scores ≥12 (*n* = 116), there was a significant main effect of baseline MASQ symptoms for each subscale (stnd beta = 0.64–0.74, *p*'s < 0.001) and a significant main effect of days since baseline on MASQ-GDA (stnd beta = −0.17, *p* = 0.0004), MASQ-AA (stnd beta = −0.08, *p* = 0.039), and MASQ-GDD (stnd beta = −0.099, *p* = 0.033). None of the other covariates or randomization group were significantly associated with any MASQ subscale (*p*'s > 0.21). Within this subgroup, there was a significant interaction effect of randomization group and days since baseline on MASQ-GDA (*p* = 0.043) and a trend toward an interaction effect of randomization group and days since baseline on MASQ-GDD (*p* = 0.097). For both subscales, there was a greater decrease over time in the CB-Abst group compared to the CB-Mon group.

## Discussion

In this study, we examined whether mood and anxiety symptoms changed during the 4 weeks following cannabis cessation among a non-clinical sample of adolescents with regular cannabis use. Given the growing number of youth that report using cannabis to cope with symptoms of anxiety and depression, it is important to understand whether mood improves or worsens with abstinence.

While we demonstrate a slight decrease in symptoms of anxiety and depression throughout the study period, this effect did not significantly differ between the abstinence and monitoring groups. This stability of mood is maintained despite increased cannabis withdrawal symptoms during the first week of abstinence. Cannabis withdrawal can include both physiological and psychological symptoms, with the most common symptoms being irritability/anger, nervousness or anxiety, decreased appetite or weight loss, restlessness, and sleep difficulties (52–54) and less common but still reported symptoms including depressed mood, stomach pain, shakiness, chills and sweating. With regard to the psychological symptoms, these onset within the first few days of abstinence and peak around 1 week from last use (52, 55). Additionally, adolescents show a lower prevalence and magnitude of withdrawal symptoms compared to adults (56). By assessing anxiety and depression symptoms for the first time at 1 week of abstinence, our study may have missed the peak of these symptoms caused by withdrawal. However, we see significantly greater withdrawal scores at 1 week after randomization in the abstinence group compared to the monitoring group but no increase in symptoms of anxiety and depression. This indicates that even if individuals experience increased depression and anxiety due to cannabis withdrawal it is likely to dissipate by the end of the first week of abstinence.

Our findings are interesting in light of the common perception among some youth cannabis users that cannabis helps treat anxiety and depression symptoms (7, 9). Mood disorders are a common reason that individuals seek medical marijuana (8). Individuals similarly use other substances, such as tobacco and alcohol, to cope with anxiety and depression. However, despite the alleviation of symptoms as a primary motive for use, cessation of use frequently benefits individuals. A definitive meta-analysis (18) reported that tobacco abstinence was associated with improved depression, anxiety, and stress, as well as positive mood and improved quality of life, with effect sizes equal to or larger than those of antidepressant medications. Other studies have shown that alcohol cessation is also associated with improved depressive symptoms (17). Again, this occurs despite self-report of people who claim that these substances improve mood and alleviate anxiety (17, 18). A previous study has shown a reduction in depression symptoms during cannabis abstinence in adults with comorbid cannabis use disorder and major depressive disorder (57). While the present study did not show improved mood symptoms after cannabis abstinence, the absence of worsening symptoms further demonstrates a conflict between people's motivations for substance use and their self-reported outcomes from cessation of use.

Our findings are in contrast to findings from Jacobus et al. (58) which demonstrate a significant reduction in depression scores but not anxiety in a group of non-treatment seeking adolescents undergoing a CM protocol, compared to a non-using control group. The current study differs from Jacobus et al. in several important ways. First, the sample size of the current study is significantly larger (*N* = 179 vs. 56). Second, the present study includes a control group of non-abstinent cannabis users who are following the same protocol (except the abstinence requirement) as the abstinent group. In the current study, we observe a decline in symptoms with abstinence, with an effect size similar to what was observed by Jacobus et al. (58); however, this change was also observed in a monitoring control group with no change in cannabis use. The decrease in symptoms over time in both the current study and the study by Jacobus et al. suggests that the effect may be better attributed to beneficial effects of participating in the study on mood symptoms and/or regression to the mean. Indeed, in Jacobus et al., there were baseline differences in mean depression scores, and it is possible that since the control group started with such low depression scores there is a floor effect such that the control group had no room to similarly decrease in their depression scores as a function of participating in the study.

While the present study is strengthened by the experimental design, randomization to abstinence, and larger sample size over previous work, the findings of this study should be viewed in the context of several limitations. First, the participants were youth engaging in recreational cannabis use and were willing and able to cease use for 4 weeks. Additionally, participants who were unable to maintain abstinence or withdrew from the study were more frequent and more severe cannabis users. Therefore, these findings may not generalize to individuals who are unable or unwilling to remain abstinent from cannabis or who are using cannabis for medical/medicinal use rather than recreational use. We were also unable to test the relative concentrations of THC or CBD in the products participants were using. It is possible that differing concentrations could have an effect on mood symptoms during abstinence. Another limitation is that mood symptoms were only assessed at weekly time points after abstinence. Since the cannabis withdrawal syndrome can begin as early as 1–2 days post-cessation and peaks around 1 week (53), we may have only caught the tail end of the period where mood symptoms are at their worst in response to withdrawal. Relatedly, we do not know the effects of longer periods of abstinence on anxiety and depression symptoms. It may take 30 days or more for cannabinoids to leave the system (41) and therefore residual cannabinoids may still be impacting the central nervous system in our current study. Finally, symptoms of anxiety and depression were assessed through self-report. It is possible that any potential level of change in these symptoms may have been too subtle for the individual to notice or that they exhibited a response bias as they were not blinded to treatment. Therefore, future studies should include clinician ratings of anxiety and depression symptoms.

In conclusion, we show that despite the common motive among adolescents of using cannabis to address mood symptoms, cannabis abstinence may not have a detrimental effect on symptoms of depression and anxiety, and may even be beneficial among adolescents who specifically report using cannabis to cope or have severe levels of use. Findings may be relevant to messaging to youth reluctant to abstain due to concerns of mood worsening. In contrast to some previous studies we do not show an significant improvement of symptoms as a function of abstinence (58, 59). This likely due to our inclusion of a matched control group of cannabis users which served to model normative fluctuations in mood within this population which further emphasizes the importance of including such control groups in experimental designs of adolescent cannabis use. Future studies will be needed to further explore the extent to which these findings translate to key subgroups, such as those with psychiatric diagnoses (cannabis use disorder, major depressive disorder, etc), and examine the effect of longer abstinence periods on these effects.

## Data Availability Statement

The raw data supporting the conclusions of this article will be made available by the authors, without undue reservation.

## Ethics Statement

The studies involving human participants were reviewed and approved by the Institutional Review Board at Massachusetts General Hospital. Written informed consent to participate in this study was provided by the participants' legal guardian/next of kin.

## Author Contributions

RS, AE, and JG contributed to the conceptualization and design of the original study. RS, JG, BT-C, and MC designed the current research question and data analyses. EL and NR were responsible for data collection. MC, BT-C, EL, and NR organized and cleaned the data. MC performed the statistical analysis and wrote the first draft of the manuscript. RS, JG, BT-C, and AE wrote sections of the manuscript. RS and AE provided funding for data collection and salary support. All authors contributed to manuscript revision, read, and approved the submitted version.

## Conflict of Interest

AE has received research grant support to her institution from Pfizer Inc, Forum Pharmaceuticals, and GSK, consultation fees from Charles River Analytics, and honoraria for advisory work from Pfizer, and Kaurna Pharmaceuticals in the past 5 years for work unrelated to this project. The remaining authors declare that the research was conducted in the absence of any commercial or financial relationships that could be construed as a potential conflict of interest.
