# Supplementary material for: Assessing Changes in Symptoms of Depression and Anxiety During Four Weeks of Cannabis Abstinence Among Adolescents
Source: Front Psychiatry. 2021 Jul 1;12:689957. doi: 10.3389/fpsyt.2021.689957 (PMC8280499; doi:10.3389/fpsyt.2021.689957)
Supplement: Supplementary file 1 [file Data_Sheet_1.docx]

Supplemental Materials

Figure S1 – Mean MASQ scores between CB-Abst and CB-Mon by visit


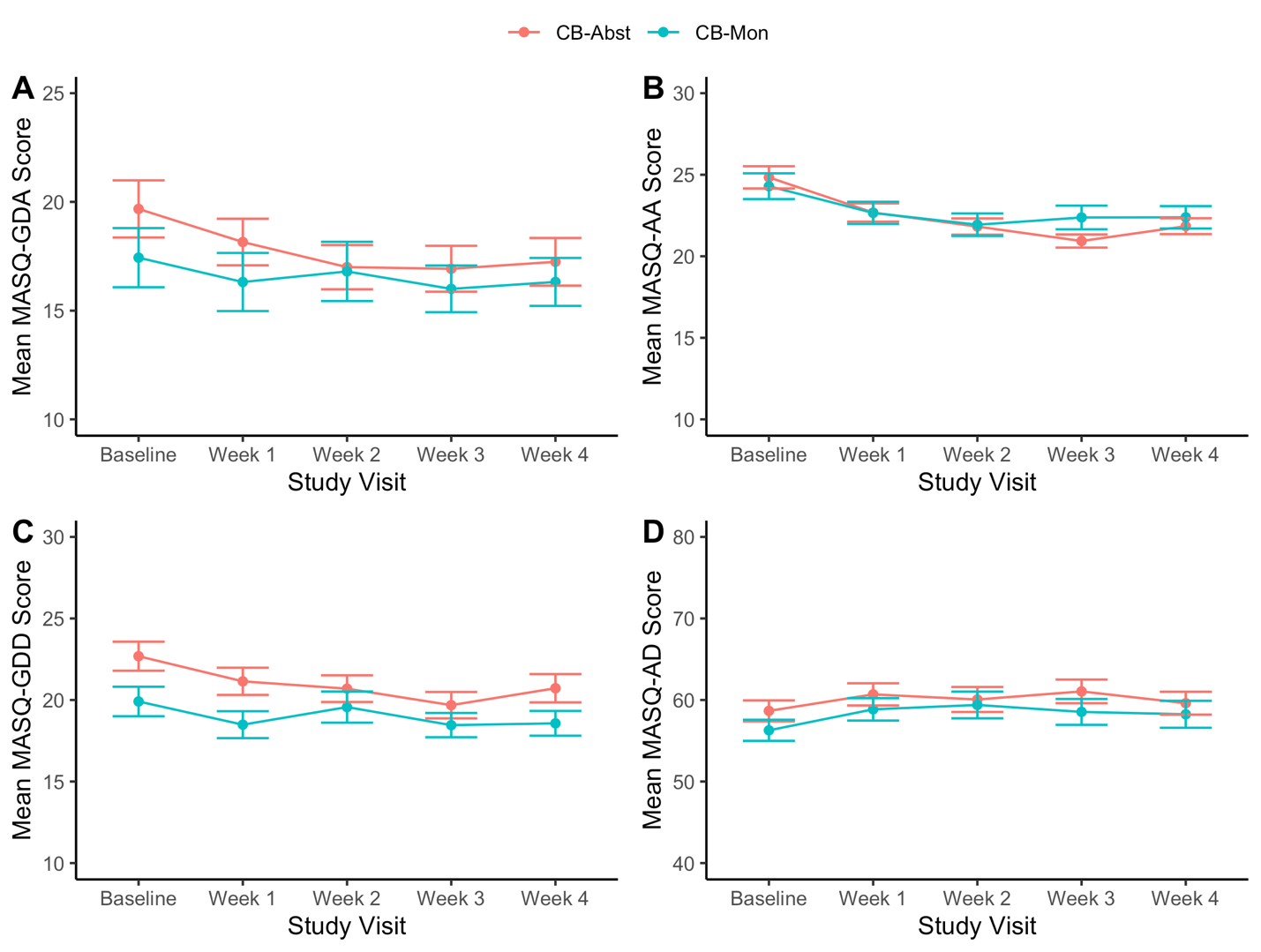


Note: Figures show the average MASQ scale with confidence intervals by group and visit with CB-Abst in coral and CB-Mon in teal.
